# Subthreshold micropulse laser for subretinal fluid after vitrectomy in myopic traction maculopathy: a pilot randomized controlled trial

**DOI:** 10.1007/s10103-026-04929-x

**Published:** 2026-06-29

**Authors:** Xifang Zhang, Haixia Ji, Zhaoyang Wang, Zi-Bing Jin, Yue Qi

**Affiliations:** 1 Beijing Tongren Eye Center, Beijing Tongren Hospital, Capital Medical University, Beijing Tongren Hospital, Beijing, China; 2 Beijing Institute of Ophthalmology, Beijing Tongren Eye Center, Beijing Tongren Hospital, Capital Medical University, Beijing Ophthalmology & Visual Science Key Laboratory, Beijing Institute of Ophthalmology, Beijing, China

**Keywords:** Myopic traction maculopathy, Subthreshold micropulse laser, Subretinal fluid, Vitrectomy

## Abstract

To evaluate the effectiveness and safety of subthreshold micropulse laser (MPL) in patients with residual subretinal fluid (SRF) after vitrectomy for myopic traction maculopathy (MTM). This was a prospective, pilot randomized controlled trial. We recruited eyes with residual SRF≥200 μm on OCT at 1-month follow-up after vitrectomy for macular hole retinal detachment (MHRD) and macular schisis with foveal detachment (MSRD). Forty eligible patients were randomized to control and MPL group in a 1:1 allocation ratio. Patients in the MPL group received MPL treatment 1-month after surgery by a same doctor and the procedure was repeated at 1-month intervals when necessary. Follow-up endpoint was defined as either complete absorption of subretinal fluid (SRF) or a minimum postoperative observation period of 6 months. The median follow-up time was 6 months (range: 3–28 months). Postoperatively, BCVA was significantly improved, and macular thickness was significantly reduced (*P* < 0.001) compared with preoperative baseline. The patients in the MPL group received 2.05 ± 0.89 (range 1–3) times of MPL. The Kaplan Meier curve showed a trend that the MPL group had better SRF absorption, but the difference is not significant between the two groups (*P* = 0.33). No severe complications in both groups during the follow-up. MPL is a safe non-invasive procedure in pathologic myopia patients. The limited therapeutic effect of MPL on SRF absorption may reflect the compromised RPE function resulting from progressive thinning and atrophy in highly myopic eyes.

## Introduction

Myopic traction maculopathy (MTM) represented by macular schisis is the main cause of visual impairment in high myopia patients. According to the atrophy (A), traction (T), and neovascularization (N) grading system (ATN) of myopic maculopathies, MTM can be categorized to six grades: no macular schisis (MS) was defined as T0, inner or outer foveoschisis was defined as T1, inner and outer foveoschisis was defined as T2, foveal detachment (MSRD) was defined as T3, full-thickness macular hole (FTMH) was defined as T4 and macular hole retinal detachment (MHRD) was defined as T5. [1, 2]MTM is more commonly seen in myopic eyes with irregular, asymmetric, or more prominent posterior staphyloma. MTM develops when the extensibility of the retina cannot adapt to the development of posterior staphyloma, under the joint force of retinal blood vessels, posterior vitreous cortex, and inner limiting membrane (ILM) [3]. MTM progresses gradually and affects visual prognosis when the integrity of the ellipsoid zone is impaired [4]. 

Therapeutically, minimal invasive vitrectomy can make the retina more elastic and easier to reattach by removing the vitreous cortex and/or the internal limiting membrane. However, in clinical observation, the absorption of the subretinal fluid after vitrectomy can take a long time, and the long-term existence of subretinal fluid affects the recovery of photoreceptor cell function and ultimately affects visual prognosis [5].

Subthreshold micropulse laser (MPL) has the biological effect of inducing retinal pigment epithelial (RPE) cell physiological function recovery by stimulating RPE cells and ultimately producing the clinical therapeutic effect of subretinal fluid absorption. It has been widely used to treat central serous chorioretinopathy, macular edema secondary to branch vein occlusion, diabetes macular edema, and other diseases, and can facilitate residual subretinal fluid absorption after vitrectomy for retinal detachment [6–9]. 

However, to date, no report has been found on the application of MPL in the residual subretinal fluid after vitrectomy for MTM. Therefore, our study intends to use MPL in the treatment of residual subretinal fluid after vitrectomy for MTM, to evaluate its effectiveness and safety.

## Methods

### Study procedures

**Fig. 1:**
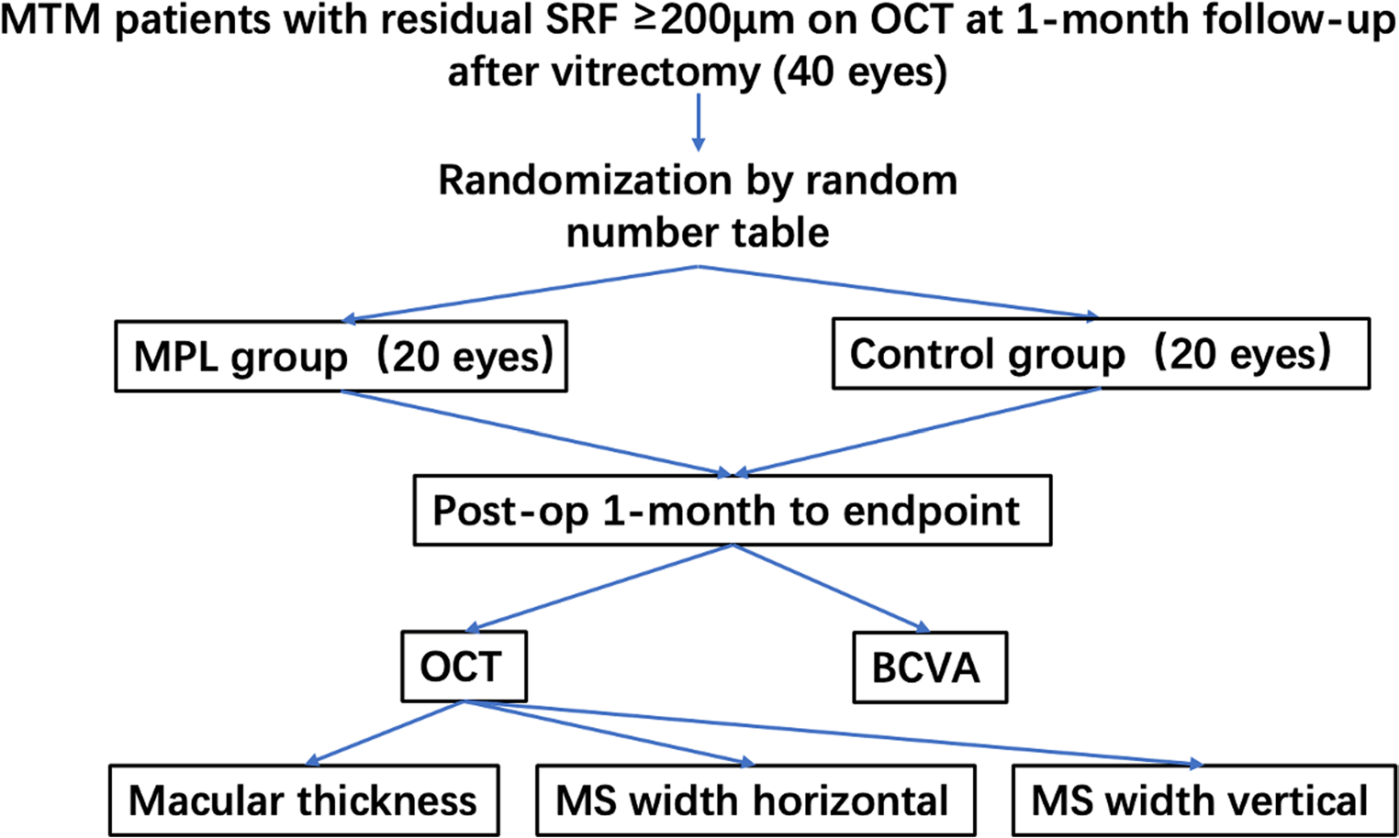
A diagram of the randomization scheme

This single-surgeon study was a prospective, pilot randomized controlled trial of eyes undergoing vitrectomy for myopic traction maculopathy from May 2022 to August 2023 at Beijing Tongren Eye Center. Inclusion criteria were patients with residual subretinal fluid ≥ 200 μm on OCT at 1-month follow-up after vitrectomy for myopic traction maculopathy. The participants were randomly assigned to the subthreshold micropulse laser or control group using a random number table. A diagram of the randomization scheme is shown in Fig. 1. Follow-up endpoint was defined as either complete absorption of subretinal fluid (SRF) or a minimum postoperative observation period of 6 months. The participants in the MPL group received MPL treatment by the same doctor and the procedure was repeated at 1-month intervals when necessary. The primary outcome was the time required for complete resolution of SRF. Secondary outcome measures included the rate of complete SRF absorption at 6 months, the mean change in best-corrected visual acuity (BCVA) from baseline, and the mean change in macular thickness.

### Trial registration

The trial was registered retrospectively at www.yxyj.org.cn/www.medicalresearch.org.cn (MR-11–24-021671) on April 29, 2024.

### Human ethics and consent to participate declarations

This study was approved by the Ethics Committee of Beijing Tongren Eye Center and adhered to the tenets of the Declaration of Helsinki (TREC2024-KY033). Written informed consent was obtained from all participants.

In both groups, BCVA was measured, and eye examinations, color fundus photographs, and OCT were performed at baseline and postoperative follow-ups until endpoint.

**Fig. 2:**
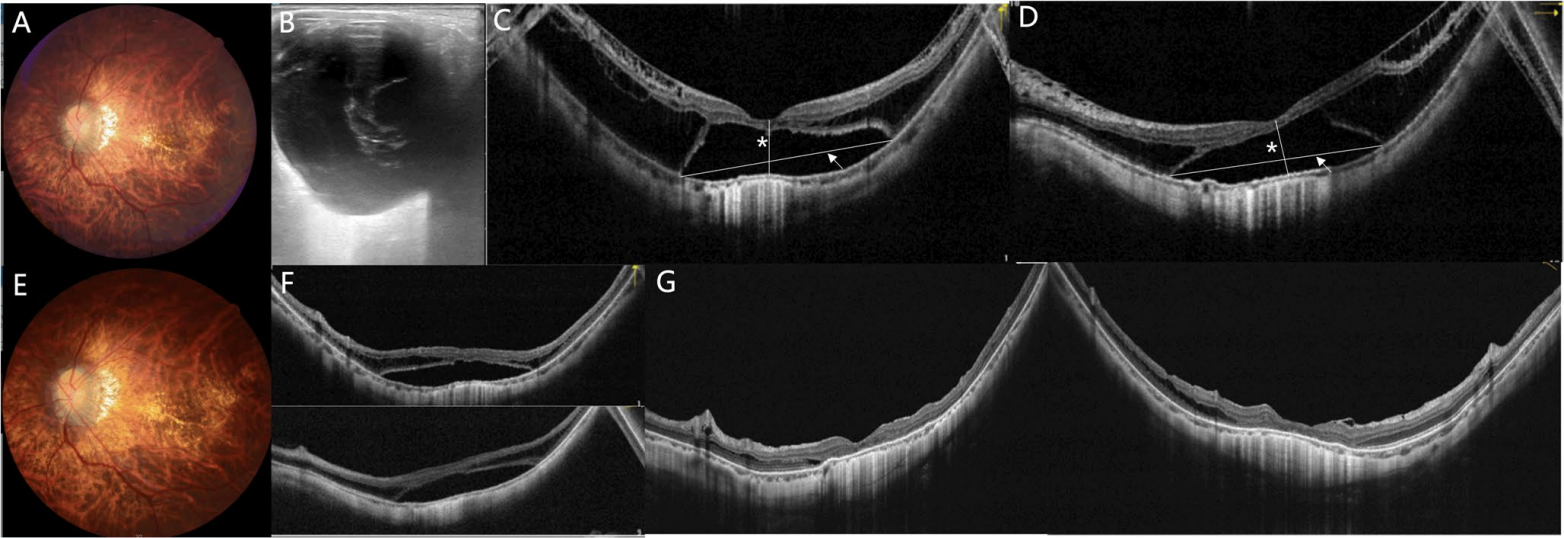
Fundoscopy and OCT from one MSRD patient in MPL group and illustration of the measurement of macular thickness, SRF width vertical and SRF width horizontal. **A** Fundoscopy showing tessellated fundus and diffuse chorioretinal atrophy before operation. **B** B-ultrasound shows prominent posterior staphyloma with AXL of 30.0 mm. **C**-**D**. Pre-op OCT presenting with MSRD and outer layer lamellar macular hole (LMH). Macular thickness was measured as the distance between ILM and RPE on vertical and horizontal OCT B-scans, and the average value was used for statistical analysis (*); SRF width vertical was measure as the width of SRF area in vertical B-scan (arrow); SRF width horizontal was measure as the width of SRF area in horizontal B-scan (arrow). **E** Fundoscopy at 1-month follow-up after PPV+ foveal-sparing ILM peeling+ phacoemulsification + intraocular lens implantation + gas tamponade surgery. **F** OCT at 1-month follow-up after surgery. MPL was conducted 3 times at 1-month, 2-month, and 4-months follow-ups. **G** Endpoint OCT showing complete SRF absorption at 7-month endpoint follow-up

The average macular thickness (the distance between ILM and RPE centered on the foveal) on the horizontal and vertical lines of the OCT radial B-scans was measured and compared between the two groups (Fig. 2). The horizontal and vertical width of subretinal fluid was also analyzed in each follow-up (Fig. 2). Sub-foveal choroidal thickness was the average value on vertical and horizontal OCT scans. BCVA after optometry was compared between the two groups. Axial length, preoperative refractive diopter, ILM peeling method, tamponade used was also recorded for each patient

### Vitrectomy

All patients received 23-gauge three-port vitrectomy with/without phacoemulsification and intraocular lens (IOL) implantation under general anesthesia by a single surgeon (Yue Qi). Posterior capsulotomy was performed at the same time. One patient in the MPL group and 2 patients in the control group was pseudophakic before surgery. One patient in each group did not underwent phacoemulsification and intraocular lens implantation due to younger age (42 yrs and 22 yrs), respectively. Laser was performed simultaneously if peripheral degeneration was noticed. ILM peeling was performed in all patients, foveal-sparing ILM peeling was performed if lamellar macular hole exists, to avoid postoperative macular hole formation. C3F8 was used as tamponade in MHRD cases, while in MSRD cases, air or C3F8 was chosen according to the surgeon’s judgment. No postoperative complication was found during the follow-up visits, such as macular hole, retinal detachment. Transit intraocular pressure rise was controlled by tropical IOP lowering eye drops and withdrawal of steroid eye drops.

### Subthreshold micropulse laser

The subthreshold micropulse laser treatment was performed with a 577-nm yellow laser system (Iridex IQ577; Laser System Iridex Corp, Mountain View, CA). Titration was performed to a barely visible burn in the midperiphery, and the treatment power was set at 50% of that level. After power titration, MPL was applied to the SRF area with a mean energy of 400 mW. Laser settings were: 200-µm spot diameter, 0.2-second exposure, 5% duty cycle, and a grid pattern (7 × 7, 5 × 5, or 3 × 3, based on lesion size) for tight coverage. If necessary, the treatment could be repeated after one month. Outcome assessors were masked to group assignment. Patients were not masked (no sham laser in control group).

### Statistical methods

#### Sample size calculation

Given the exploratory nature of this study and the lack of prior data on subthreshold MPL efficacy for residual SRF in MTM patients, a formal a priori sample size calculation was not performed. This pilot trial aimed to enroll 20 patients per group to estimate effect sizes and safety, based on clinical feasibility and consistency with previous studies on similar interventions [2, 3], and no formal power calculation was conducted. With 20 patients per group and observed resolution rates of 70% versus 55%, the post hoc power was 38% at a significance level of α = 0.05.

### Data analysis

Data analysis was performed using SPSS 22.0 software. The Shapiro-Wilk test was applied to assess data normality. Continuous variables with non-normal distribution were expressed as median (interquartile range), while normally distributed data were presented as mean± standard deviation. Snellen visual acuity was converted to LogMAR BCVA for statistical analysis.

Demographic and clinical characteristics at baseline and endpoint between groups were compared using independent-samples t-test, χ² test, and Kruskal-Wallis nonparametric test as appropriate. All statistical tests were two-tailed, with a P-value < 0.05 considered statistically significant. A 95% confidence interval was adopted for all analyses. The Kaplan Meier curve was performed by www.empowerstats.com and R software.

## Results

### Demographic and baseline characteristic

At baseline, there were significant differences between the two groups in age (*p* < 0.001) and sex distribution (*p* < 0.001). No significant differences were observed in any of the other baseline variables, including diagnosis, follow-up time, ILM peeling, tamponade, axial length (AXL), diopter, BCVA, macular thickness, horizontal SRF width, vertical SRF width, and choroidal thickness (Table 1).

**Table 1:** Demographic and baseline characteristic

Parameter	MPL group	Control group	*p* Value
Age in mean ± SD	52.99 ± 6.83	55.49 ± 12.46	= 0.128†
Sex, no. of patients			= 0.465*
Male	6	4	
Female	14	16	
Diagnosis by eye, n			= 0.881*
MSRD	16	16	
MHRD	4	4	
Median follow-up duration in months (range)	6(3–28)	5.5(3–22)	0.784‡
ILM peeling			0.478*
Without foveal sparing	11	8	
With foveal sparing	9	12	
tamponade			0.793*
air	12	10	
C3F8	8	10	
Diopter, D	−12.50 (−15.25, −7.38)	−15.00 (−15.62, −10.88)	0.513‡
Axial length, mm	30.18 ± 1.44	29.72 ± 1.63	0.414†
BCVA (in LogMar)	0.91(0.70–1.30)	0.70(0.49–0.87)	0.055‡
Macular thickness, µm	594.40 ± 183.35	582.00 ± 250.02	0.865†
SRF width horizontal, µm	3250.00(2047.50–3747.50)	2170.00(1556.50–4357.50)	0.417‡
SRF width vertical, µm	2880.00(2150.00–3646.00)	2030.00(1390.00–4505.00)	0.474‡
Choroidal thickness, µm	45.90 ± 19.91	51.56 ± 25.12	0.455†

*χ2 test

†Independent-samples t test, results were show as mean ± SD

‡ Kruskal-Wallis nonparametric test, results were show as median (IQR)

### Endpoint result

At the endpoint, 14 eyes (70%) in the MPL group and 11 eyes (55%) in the control group showed complete SRF absorption (Table 2), with an absolute difference of 15% (95% CI could not be calculated due to the small sample size). The endpoint BCVA and macular thickness were significantly improved compared to baseline (*P* < 0.001). There was no significant difference in the endpoints BCVA (*P* = 0.939) and macular thickness (*P* = 0.143) between the two groups (Table 3).

**Table 2:** The follow-up time and percent of eyes with complete SRF absorption in two groups

post-op	3 m	6 m	9 m	12 m	Endpoint
MPL (20 eyes)	3(15%)	6(30%)	3(15%)	2(10%)	14(70%)
Control (20 eyes)	2(10%)	2(10%)	4(20%)	3(15%)	11(55%)
Total (40 eyes)	5(12.5%)	8(20%)	7(17.5%)	5(12.5%)	25(62.5%)

**Table 3:** Endpoint parameters and comparison with baseline

Parameter	MPL group	Control group	*p* Value
endpoint (%)			0.967*
No SRF	14 (70.0)	11 (55.0)	
Residual SRF	6 (30.0)	9 (35.0)	
BCVA (in LogMar, mean (IQR))	0.30 [0.22, 0.52]	0.40 [0.26, 0.52]	0.939‡
Baseline BCVA (mean (IQR))	0.91(0.70–1.30)	0.70(0.49–0.87)	< 0.001
macular thickness, µm, mean (SD)	211.67 (56.32)	172.91 (56.18)	0.143†
Baseline macular thickness, µm	594.40 ± 183.35	582.00 ± 250.02	< 0.001

*χ2 test

†Independent-samples t test

‡ Kruskal-Wallis nonparametric test, results were show as median (IQR)

**Fig. 3:**
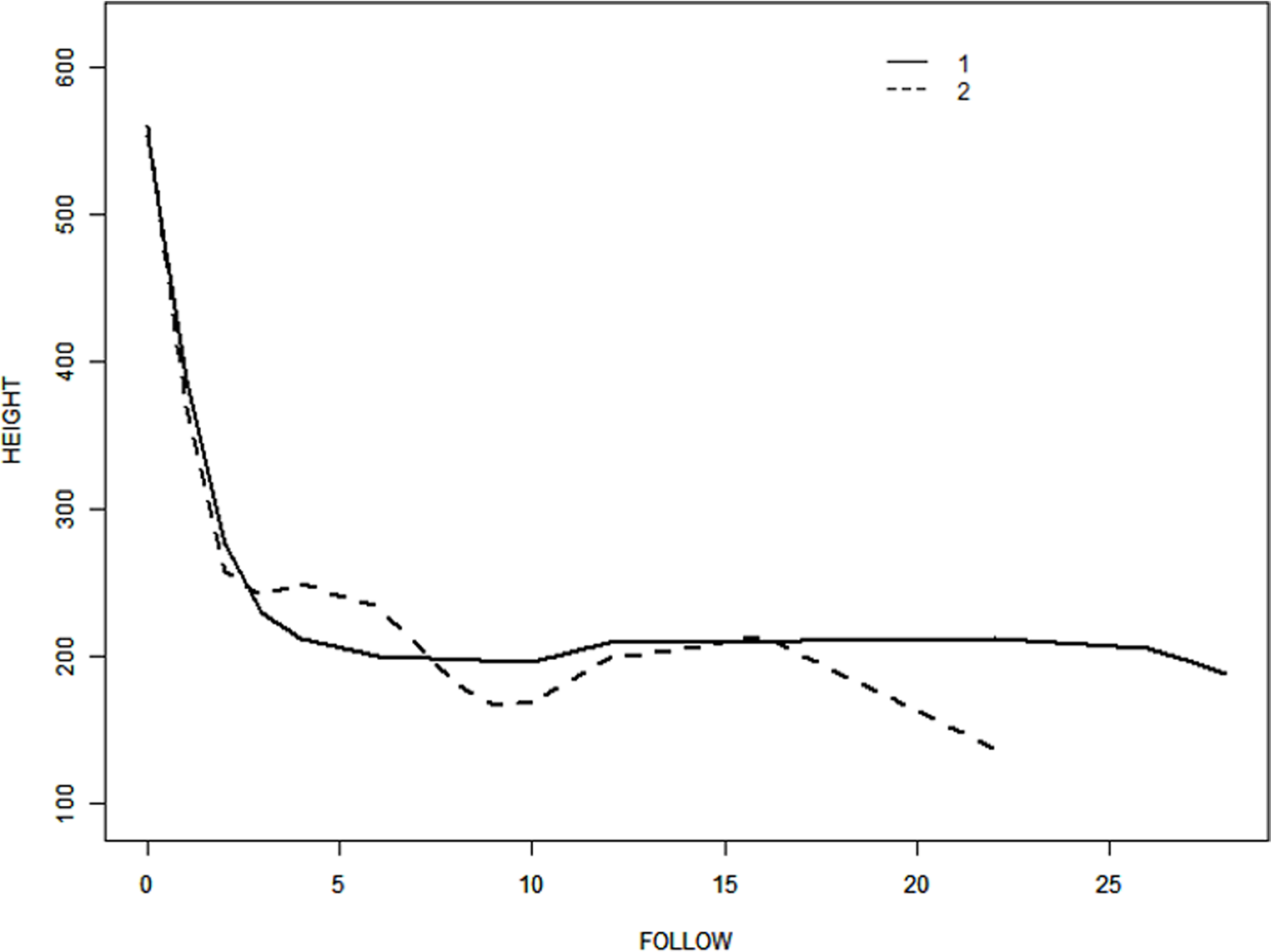
Macular thickness (µm) decreases with prolonged time. A more stable curve was seen in MPL group (Group 1: MPL group; Group 2: control group)

**Fig. 4:**
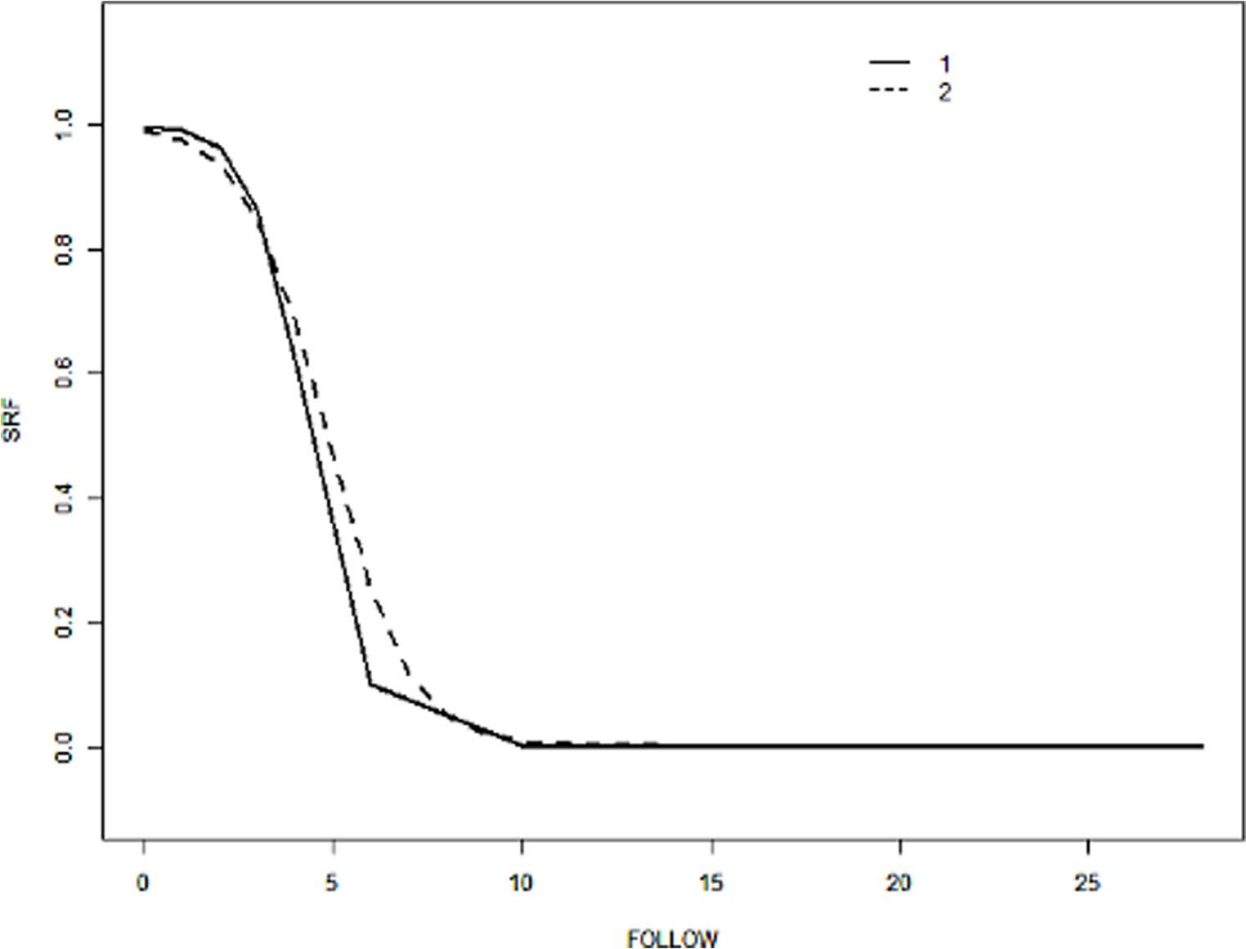
The percent of eyes with complete SRF absorption was increasing with prolong time in both groups (Group 1: MPL group; Group 2: control group)

**Fig. 5:**
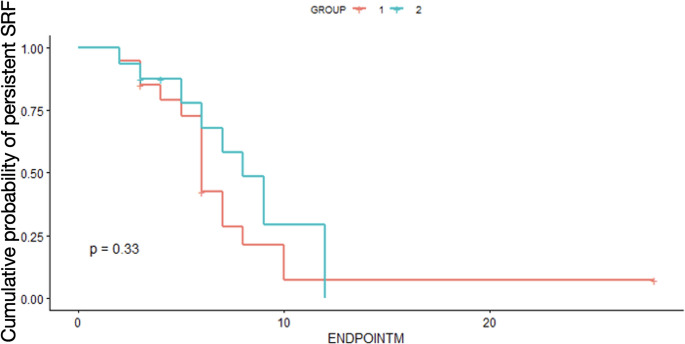
Kaplan-Meier curve comparing the probability of persistent SRF between the MPL group and control group. No significant difference was found (log-rank test, *P* = 0.33). (Group 1: MPL group; Group 2: control group)

The patients in the MPL group received 2.05 ± 0.89 (range 1–3) times of MPL. Macular thickness decreases with prolonged time. Figure 3 showing a more stable curve in MPL group. The percent of eyes with complete SRF absorption was increasing with prolong time in both groups (Fig. 4). The median follow-up time was 6 months (range: 3–22 months). The Kaplan Meier curve showed no significant difference in survival curves between the two groups (*P* = 0.33). However, The MPL group showed better SRF absorption (Fig. 5)

## Discussion

Prolonged SRF persistence following MTM surgery represents a common clinical scenario, with resolution potentially requiring 1–2 years. Prolonged post-op SRF duration correlates with poorer BCVA at 12 months follow-up [5, 10]. This motivated our investigation into whether subthreshold MPL could enhance SRF absorption through RPE stimulation, thereby improving visual outcomes.

The 577-nm yellow subthreshold micropulse laser is an innovative retinal laser therapy that delivers controlled, ultrashort pulse trains at subvisible energy levels to induce therapeutic biological effects while avoiding thermal tissue damage. This unique photobiomodulation effect promotes fluid resorption in various chorioretinal disorders, including central serous chorioretinopathy and intraretinal fluid in various conditions including diabetic macular edema, postoperative cystoid macular edema and other miscellaneous conditions [11]. However, its effect in reducing SRF in myopic traction maculopathy has not been investigated.

Our study demonstrates that MPL exhibits a certain, albeit non-significant, effect in reducing SRF following vitrectomy for MTM. Given the low statistical power (38%), this pilot study cannot rule out a clinically meaningful benefit of the intervention. The observed absolute difference of 15% in resolution rates suggests that a larger, definitively powered trial is warranted, with an estimated sample size of 80–100 patients per group. Therefore, our findings should be considered preliminary and hypothesis-generating rather than conclusive.

The pathogenesis of preoperative SRF in MTM is likely multifactorial, with mechanical traction being a predominant contributing factor. For instance, the pathogenesis of subretinal fluid (SRF) in dome-shaped macula likely involves mechanical traction forces. These traction-induced structural alterations create potential spaces that facilitate fluid accumulation, while concurrently promoting elevated cytokine levels within these spaces, ultimately leading to SRF formation [12]. The RPE pump function in myopic traction maculopathy (MTM) may become compromised due to chronic mechanical traction/compression and persistent ischemia, leading to impaired fluid transport capacity [13]. When coexisting with outer lamellar holes, structural disruption of the retina-choroid interface may impair fluid absorption, thereby facilitating SRF accumulation. Furthermore, in cases of macular hole retinal detachment, the SRF composition may include liquefied vitreous components.

Following vitrectomy for MTM, the removal of both the posterior vitreous cortex and internal limiting membrane relieves tractional forces, leading to gradual resolution of SRF. This observation strongly implicates mechanical traction as a principal pathogenic factor in SRF accumulation. However, the slow absorption of subretinal fluid (SRF) following surgery in MTM patients may be attributed to other factors besides traction: (1) The traction in MTM develops gradually over time, resulting in prolonged SRF persistence and consequently slower absorption. (2) Impaired RPE pump function in MTM patients. Additionally, studies have reported that pathologic myopia patients exhibit significant choroidal thinning in the macular region, yet demonstrate compensatory increased blood flow that elevates local hydrostatic pressure, thereby promoting fluid leakage into the subretinal space [14]. (3) Vitrectomy itself may induce postoperative elevation of inflammatory factors, which could also contribute to SRF accumulation.

The rationale for attempting sub-threshold micropulse laser (MPL) therapy for persistent postoperative subretinal fluid (SRF) lies in its unique capacity to safely activate retinal pigment epithelium (RPE) cells and intraretinal Müller cells while avoiding visible retinal damage. Its mechanism of action involves stimulating heat-shock protein production-highly conserved molecular chaperones that protect cells by inhibiting apoptotic and inflammatory pathways [11].

Previous studies indicate that highly myopic patients undergoing scleral buckling or vitrectomy for rhegmatogenous retinal detachment exhibit delayed SRF resolution compared to normal-axial-length eyes. Increased axial length constitutes an independent risk factor for prolonged SRF persistence, potentially attributable to RPE/choriocapillaris loss in atrophic zones impairing fluid transport mechanisms. The severity of choroidal atrophy inversely correlates with SRF absorption rates [15].

In our study, all patients were T3-T5 stage, presenting with extreme myopia and posterior pole chorioretinal atrophy (either diffuse or patchy, as illustrated in Fig. 2). A potential explanation for our inconclusive result may involve the low RPE pigmentation and progressive RPE thinning/atrophy commonly seen in highly myopic eyes, although RPE integrity was not directly quantified. These factors reduce laser energy absorption and compromise RPE function, thereby limiting any biological effect on SRF absorption.

The MPL protocol was designed to deliver treatment in multiple minimal-dose sessions, with patients in the MPL group receiving an average of 2.05 ± 0.89 treatments (range 1–3). Notably, no cases of secondary choroidal neovascularization were observed, demonstrating the safety profile of MPL for pathologic myopia patients. In highly myopic eyes with posterior staphyloma, the retinal surface at the staphyloma floor is farther from the cornea than the titration site (typically the vascular arcades). This results in a larger actual laser spot diameter at the treatment area due to transverse magnification, leading to a lower power density (irradiance) than intended. For an eye with an axial length of 30 mm, the actual spot diameter increases by 15–20%, reducing irradiance. Consequently, the target area may have been undertreated, which could partially explain the limited therapeutic effects observed in this study. Future studies should consider adjusting laser parameters based on individual axial length and staphyloma depth.

Other potentially beneficial treatment options for subretinal fluid (SRF) may include oral carbonic anhydrase inhibitors, while surgical approaches could involve silicone oil tamponade or combined posterior scleral reinforcement procedures [15–18]. We opted for gas tamponade primarily because highly myopic patients are particularly susceptible to secondary glaucoma following silicone oil tamponade [19]. Gas tamponade effectively avoids this postoperative complication while providing stronger surface tension that enables effective pressure on macular [19–21]. In our previous research, we have performed vitrectomy combined with posterior scleral reinforcement for MTM patients [15, 22]. However, due to limited availability of allogeneic scleral materials, this approach was not utilized in the current study.

Several limitations should be acknowledged in this preliminary research. The primary limitation of this study is the small sample size and corresponding low statistical power, which may have underestimated the true treatment effect. Larger sample size studies are needed to determine whether MPL confers significant benefit. Secondly, autofluorescence imaging could potentially help evaluate whether subthreshold MPL caused irreversible damage [23, 24]. However, interpretation may be confounded in our MTM patients, since all patients presented with posterior staphyloma and varying degrees of posterior pole retinal-choroidal atrophy. Future studies should assess RPE and choroidal integrity using advanced imaging techniques to determine whether MPL exerts a genuine biological effect beyond the small beneficial trend observed in this study. Thirdly, future studies could incorporate comparative analysis with silicone oil tamponade to determine its potential advantage in promoting SRF absorption. Finally, further analysis is warranted to analyze factors influencing SRF resolution, including refractive error, axial length, tamponade selection, internal limiting membrane peeling method and choroidal thickness.

In conclusion, our preliminary findings demonstrate that subthreshold MPL therapy represents a safe, non-invasive adjunctive treatment for residual SRF in MTM patients. Despite its excellent safety profile, MPL failed to significantly enhance postoperative SRF resolution in MTM eyes, likely due to underlying RPE thinning and functional impairment associated with pathologic myopia. Given its safety and the lack of better alternatives, it should be reserved for carefully selected cases after thorough discussion of uncertain benefits.

## Data Availability

The raw data supporting the conclusions of this article will be made available by the authors, without undue reservation.
